# Genome mining, antibacterial activity, and metabolomic profiling reveal the biosynthetic potential of *Burkholderia glumae* isolates from Thailand with activity against clinically important bacterial pathogens

**DOI:** 10.1371/journal.pone.0361711

**Published:** 2026-10-30

**Authors:** Sujin Patarapuwadol, Panalee Oupaijit, Pornpavee Nualnisachol, Peerut Chienwichai, Jiraphan Premsuriya

**Affiliations:** 1 Department of Plant Pathology, Faculty of Agriculture at Kamphaeng Saen, Kasetsart University Kamphaeng Saen Campus, Nakhon Pathom, Thailand; 2 Princess Srisavangavadhana Faculty of Medicine, Chulabhorn Royal Academy, Bangkok, Thailand; 3 Research Center on Clinical and System Microbiology (RCSyM), Chulabhorn Royal Academy, Bangkok, Thailand; Cardiff’s Metropolitan University: Cardiff Metropolitan University, UNITED KINGDOM OF GREAT BRITAIN AND NORTHERN IRELAND

## Abstract

The increasing prevalence of antimicrobial resistance has underscored the need for novel antimicrobial agents. Members of the genus *Burkholderia* produce a diverse array of antimicrobial metabolites; however, the biosynthetic potential of *Burkholderia glumae*, a plant pathogen, remains relatively underexplored. In this study, we investigated the biosynthetic potential, antibacterial activity, and metabolomic profiles of 16 *B. glumae* isolates collected from Thailand. Genome mining using antiSMASH and DeepBGC identified 14–17 and 40–51 biosynthetic gene clusters (BGCs) per genome, respectively. Multiple conserved BGC classes were detected across all isolates, including terpene-, polyketide-, and nonribosomal peptide-associated clusters. DeepBGC further predicted that 22–32 BGCs per isolate were associated with antibacterial activity. Phenotypic screening using soft-agar overlay assays on LB agar demonstrated that all 16 isolates displayed antibacterial activity against clinically important bacterial pathogens, including *Acinetobacter baumannii*, *Escherichia coli*, and *Staphylococcus aureus*. Four isolates exhibiting the strongest antibacterial activity were selected for further metabolomic analysis. Untargeted LC–MS/MS profiling of extracts from inoculated and uninoculated LB agar plates identified 68 putatively annotated antimicrobial-related metabolites shared among all four isolates and significantly enriched relative to the uninoculated controls (fold change > 2, *p* < 0.05). These metabolites belonged to diverse chemical classes, including macrolactams, triterpene saponins, triterpenoids, hydroxyquinolones, phenolic glycosides, and cyclic depsipeptides. Macrolactams represented the most frequently detected metabolite class, whereas triterpene saponins exhibited the highest enrichment relative to control LB agar without bacterial inoculation. Toxoflavin, reumycin, and bactobolin A were not detected in extracts from cultures grown on LB agar at 37 °C for 24 h. These findings, which demonstrate that *B. glumae* isolates from Thailand possess diverse biosynthetic capabilities and antibacterial activity against clinically important bacterial pathogens, highlight the potential of these isolates as a source of bioactive metabolites for future efforts in antimicrobial agent discovery.

## Introduction

Antimicrobial resistance has emerged as a critical global health challenge. Past estimates have indicated that approximately 4.95 million deaths worldwide were associated with bacterial antimicrobial resistance in 2019 [1], and current projections suggest that annual deaths associated with antimicrobial resistance could exceed 8.22 million by 2050 if effective interventions are not implemented [2]. Particularly concerning are the ESKAPEE pathogens (*Enterococcus faecium, Staphylococcus aureus, Klebsiella pneumoniae, Acinetobacter baumannii, Pseudomonas aeruginosa, Enterobacter* spp. and *Escherichia coli*), which account for a significant proportion of multidrug-resistant healthcare-associated infections worldwide [3]. The remarkable capacity of these pathogens to acquire and disseminate resistance determinants has severely compromised the effectiveness of existing antibiotics, creating an urgent need for novel antimicrobial agents [4–6]. However, the slowing of antibiotic discovery pipelines due to scientific, economic, and regulatory challenges is now highlighting the importance of exploring alternative sources of antimicrobial compounds [7].

Microorganisms remain one of the most important sources of clinically useful antibiotics [8] because they naturally produce diverse specialized metabolites with potent antimicrobial activities that provide competitive advantages in complex microbial communities [9]. In this context, species of the genus *Burkholderia* have gained attention as producers of structurally diverse specialized metabolites [10,11]. Notably, comparative genomic studies have shown that several *Burkholderia* species possess extensive biosynthetic gene cluster (BGC) repertoires, with BGCs accounting for more than 10% of the genome in some species [10,12,13]. These gene clusters encode a wide range of structurally diverse specialized metabolites, including nitrogen-containing heterocycles, volatile organic compounds, polyenes, polyynes, siderophores, macrolides, bacteriocins, quinolones, and other unclassified metabolites [14]. Notably, several of the metabolites produced by *Burkholderia* species have demonstrated therapeutic antimicrobial potential. Examples include cepacins, which display potent activity against staphylococci [15]; thailandamide and thailandenes, which inhibit bacterial growth through interference with fatty acid biosynthesis [16]; gladiolin, a macrolide antibiotic active against *Mycobacterium tuberculosis* [17]; and 4-hydroxy-2-alkylquinolines, which contribute to interbacterial competition and antimicrobial activity [18]. Additional compounds, such as bactobolins and phenazine derivatives, also reflect the extensive chemical diversity found within this genus [12]. The structural novelty and diverse mechanisms of action of these metabolites suggest that *Burkholderia* species represent a promising reservoir of compounds that may help address the growing challenge of antimicrobial resistance.

Among members of the *Burkholderia* genus, *B. glumae* remains relatively underexplored as a source of antimicrobial compounds. This species is primarily recognized as a plant pathogen that causes bacterial grain rot in rice [19]. Despite the importance of this species as a plant pathogen, several studies have highlighted its potential as a producer of antimicrobial metabolites. For example, a pyrazole-derived compound isolated from *B. glumae* demonstrated antibacterial activity against another plant pathogen, *Erwinia amylovora* [20]. Antimicrobial activities against clinically relevant bacterial pathogens, including *S. aureus* and *A. baumannii*, have also been reported [21]. Analysis of active isolates using semipreparative fractionation and liquid chromatography–mass spectrometry (LC–MS) has also identified several known antimicrobial metabolites, including reumycin, toxoflavin, and bactobolin A, as well as two putatively novel compounds with the molecular formulas C_16_H_23_C_l2_N_3_O_6_ and C_16_H_22_C_l2_N_2_O_7_ [21]. However, some of these metabolites, particularly the toxoflavin-related compounds, have known cytotoxic properties that may limit their therapeutic applicability [22–24].

At present, studies investigating the antimicrobial potential of *B. glumae* remain limited and have focused on a relatively small number of isolates. The genomic basis underlying specialized metabolite production in *B. glumae* also remains incompletely characterized, particularly with respect to the diversity of BGCs and their relationship to antimicrobial metabolite production. Consequently, additional studies that integrate genome mining, antibacterial screening, and metabolomic analyses are needed to obtain a better characterization of the antimicrobial potential of *B. glumae*.

In this context, recent advances in genome mining and metabolomics are now providing powerful tools for natural product discovery [25]. Genome-based approaches enable the identification of BGCs that may encode previously unknown specialized metabolites, while LC–MS-based metabolomics facilitates the comprehensive profiling of metabolites produced under specific growth conditions [25,26]. In the present study, we explored the possibility of integrating genomic and metabolomic analyses of *B. glumae* isolates to accelerate the identification of candidate bioactive compounds and provide insights into the biosynthetic potential of this microorganism. To do this, we investigated the biosynthetic potential, antibacterial activity, and metabolomic profiles of 16 *B. glumae* isolates collected in Thailand. We then explored the predicted BGCs using genome mining, and we evaluated antibacterial activity against clinically relevant bacterial pathogens. Our subsequent LC–MS/MS-based metabolomic profiling of the most active isolates identified metabolites associated with antibacterial activity. Collectively, our findings provide new insights into the antimicrobial potential of *B. glumae* isolates from Thailand and highlight their value as a source of candidate metabolites for future antimicrobial discovery efforts.

## Materials and methods

### Bacterial strains

*B. glumae* isolates, which had been collected from rice fields in seven provinces of Thailand between 2011 and 2017, were obtained from the Department of Plant Pathology, Faculty of Agriculture at Kamphaeng Saen, Kasetsart University, Thailand. Whole-genome sequence data and genomic characteristics of these isolates, which have been reported previously [27], were used in the present study for the BGC analysis. *A. baumannii* ATCC 19606, *E. coli* ATCC 25922, *S. aureus* ATCC 29213, *K. pneumoniae* ATCC 700603, and *P. aeruginosa* ATCC 27853 were obtained from the Central Laboratory, Chulabhorn Hospital, Bangkok, Thailand. Before storage, each bacterial isolate was cultured on Luria–Bertani (LB) agar, Miller (Difco, Becton, Dickinson and Company, USA). Colonies were collected directly from the agar surface, suspended in sterile 20% (v/v) glycerol solution, and stored at −80 °C until use.

### Genome mining for specialized metabolite BGCs in *B. glumae*

The genomic mining tools antiSMASH [28] and DeepBGC [29] were used with default parameters to analyze the genome assemblies of the 16 *B. glumae* isolates (BioProject accession: PRJNA1307573) for BGCs associated with specialized metabolite production. The antiSMASH tool was used to identify and annotate putative BGCs based on sequence similarity to known specialized metabolite biosynthetic pathways, whereas DeepBGC was used to predict BGCs and classify their product classes and potential biological activities using machine learning. The presence of toxoflavin biosynthetic genes was investigated independently of antiSMASH using BLASTn, with the toxoflavin biosynthesis-associated locus spanning *toxJ* to *toxE* from *B. glumae* BGR-1 (NCBI accession: NC_012721.2) [30] as the reference query.

### Screening of the antibacterial activity of *B. glumae* isolates

The antibacterial activity of *B. glumae* isolates was assessed using a soft-agar overlay assay adapted from a previously described method [31]. Briefly, *B. glumae* isolates were recovered from −80 °C glycerol stocks and streaked onto LB agar, followed by overnight incubation at 37 °C. A single colony was then inoculated into 10 mL LB broth and incubated overnight at 37 °C with shaking at 150 rpm. Subsequently, 5 µL of the overnight culture was spotted onto LB agar plates, allowed to dry, and incubated at 37 °C for 24 h. Indicator strains, including *A. baumannii* ATCC 19606, *E. coli* ATCC 25922, *S. aureus* ATCC 29213, *K. pneumoniae* ATCC 700603, and *P. aeruginosa* ATCC 27853, were prepared by streaking onto LB agar plates and incubating overnight at 37 °C. A single colony of each strain was inoculated into 10 mL LB broth and grown overnight at 37 °C with shaking at 150 rpm. The overnight cultures were then diluted 1:100 in 10 mL fresh LB broth and incubated under the same conditions until mid-log phase (OD600 ≈ 0.5). For the overlay assay, 80 µL of each indicator culture was added to 8 mL of sterile molten soft agar (0.8% [w/v] bacteriological agar in deionized water) maintained at 42 °C, gently mixed, and poured over the agar plates containing the pre-grown *B. glumae* spots. The plates were incubated overnight at 37 °C. Antibacterial activity was determined by the presence of clear zones surrounding the *B. glumae* colonies. The clear zone sizes were measured in millimeters.

### Agar plug assay for antibacterial activity

The antibacterial activity of selected *B. glumae* isolates was also evaluated using an agar plug assay [32]. Indicator strains were prepared as described above and incorporated into soft-agar overlay plates. Briefly, overnight cultures of each indicator strain were diluted 1:100 in 10 mL LB broth and grown to mid-log phase (OD_600_ ≈ 0.5). An aliquot (80 µL) of each culture was mixed with 8 mL of sterile molten soft agar prepared as described above, maintained at 42 °C and overlaid onto LB agar plates. Selected *B. glumae* isolates were prepared as described above, except that 12 spots of each isolate were inoculated per LB agar plate and incubated at 37 °C for 24 h. Following incubation, agar plugs (5 mm diameter) were excised from the agar areas surrounding the colony spots and carefully transferred onto the surface of the indicator strain overlay plates. The plates were incubated at 37 °C overnight, and antibacterial activity was assessed by the presence of clear zones surrounding the agar plugs.

### LC–MS/MS-based metabolite profiling

Selected *B. glumae* isolates were cultured on LB agar plates as described for the agar plug assays. After incubation, bacterial colonies were removed by washing the agar surface with sterile deionized water, and the agar was collected and mechanically ground into small pieces to facilitate metabolite extraction. Four agar plates (20 mL agar per plate) were pooled to constitute one sample (80 mL total agar volume), and three biological replicate samples were prepared for each *B. glumae* isolate for subsequent analysis. Uninoculated LB agar plates prepared without bacterial inoculation and incubated under the same conditions were used as medium controls and subjected to the same collection, extraction, and LC–MS/MS procedures as the inoculated samples.

Agar samples were extracted with 80 mL of 100% methanol and sonicated for 30 min, following a protocol adapted from Depoorter et al. [21]. Extracts were centrifuged at 5,000 rpm for 10 min, and the supernatants were collected and evaporated to dryness overnight at 40 °C. The samples were lyophilized, weighed, and reconstituted to a final concentration of 20 mg/mL using 70% methanol containing 50 ng/mL sulfadimethoxine as an internal standard. A pooled quality control (QC) sample was prepared by combining 50 µL of each sample. Dilution QC samples of 0%, 1%, 10%, 20%, 50%, and 80% were also prepared from the pooled QC. All samples and QC samples were centrifuged at 14,000 rpm for 5 min, and the supernatants were transferred to LC vials for analysis.

LC–MS/MS analysis was performed using an Agilent LC-QTOF 6545XT system (Agilent Technologies, USA) in both positive and negative high-resolution ionization modes. Chromatographic separation was carried out on a Poroshell 120 EC-C18 column (2.1 × 100 mm, 2.7 µm) (Agilent Technologies, USA) maintained at 50 °C, with an injection volume of 10 µL. Mobile phase A consisted of 0.1% formic acid in water, and mobile phase B consisted of 0.1% formic acid in acetonitrile, delivered at a flow rate of 0.4 mL/min. The gradient elution program was as follows: 0% B for 0.5 min (0.0–0.5 min), 0–55% B over 10.0 min (0.5–10.5 min), 55–75% B over 2.0 min (10.5–12.5 min), 75–100% B over 1.5 min (12.5–14.0 min), 100% B for 3.0 min (14.0–17.0 min), and 100–0% B over 0.5 min (17.0–17.5 min), followed by re-equilibration at 0% B for 2.5 min (17.5–20.0 min).

The mass spectrometer was operated in high-resolution data-dependent acquisition using both positive and negative ESI modes. The drying gas temperature was 325 °C at a flow rate of 13 L/min, the sheath gas temperature was 275 °C at 12 L/min, and the nebulizer pressure was 45 psi. Capillary voltages were set to 4,000 V in positive mode and 3,000 V in negative mode. MS spectra were acquired over an m/z range of 40–1700, and MS/MS spectra were acquired over an m/z range of 25–1000 with an acquisition rate of 3.35 spectra/s. A maximum of 10 precursor ions per acquisition cycle was selected for fragmentation using collision energies of 20 eV (positive mode) and 10 eV (negative mode), with a precursor intensity threshold of 5,000 counts. Continuous mass calibration was performed using reference masses of m/z 121.0509 and 922.0098 in positive mode and m/z 112.9856 and 1033.9881 in negative mode.

## Metabolite annotation and identification

Raw LC–MS/MS data were processed using MS-DIAL version 5.3 [33]. Peak alignment was performed against the QC R3 sample. Data were normalized using the sulfadimethoxine internal standard and the LOWESS method, and reference mass ions used for continuous mass calibration were excluded from downstream analysis. Metabolite annotation was performed by matching accurate mass, retention time, and MS/MS spectra against the MS-DIAL ESI (+/−) MS/MS authentic standards database using [M + H]⁺ and [M − H]^−^ as the default adducts for positive and negative ionization modes, respectively. Putative metabolite annotations were accepted using an identification score of ≥ 0.70 and a mass error threshold of ≤ 25 ppm. Features with retention times outside 0.2–18 min, Pearson correlation coefficients < 0.70 against the dilution QC series, or QC coefficients of variation (CV) > 50% were excluded from further analysis. The processed LC–MS/MS feature table, including putative metabolite annotations and peak intensities for all samples, is provided in S1 Table.

## Statistical analysis of metabolomics data

Statistical analyses of metabolomics data were conducted using the MetaboAnalyst 6.0 platform [34] using the One Factor module. Prior to analysis, the metabolomic features were filtered based on the interquartile range using median peak intensity values to remove features with low variability. The data were subsequently normalized by quantile normalization, transformed using the cube root transformation, and scaled by range scaling. Principal component analysis (PCA) was performed to evaluate the overall variation among samples and to assess analytical reproducibility. QC samples were included in the PCA, and analytical reproducibility was assessed according to the clustering of QC samples. For each *B. glumae* isolate, metabolites with significantly increased abundance were identified based on a fold change >2 and *p* value <0.05 relative to uninoculated LB agar controls.

## Results

### BGCs predicted in *B. glumae* genomes

Genome mining using antiSMASH and DeepBGC revealed that all 16 *B. glumae* isolates showed clear biosynthetic potential (Fig 1, S1–S2 Tables). The antiSMASH tool identified 14–17 BGCs per genome, whereas DeepBGC predicted 40–51 putative BGCs per genome (Fig 1A and 1B).

**Fig 1:**
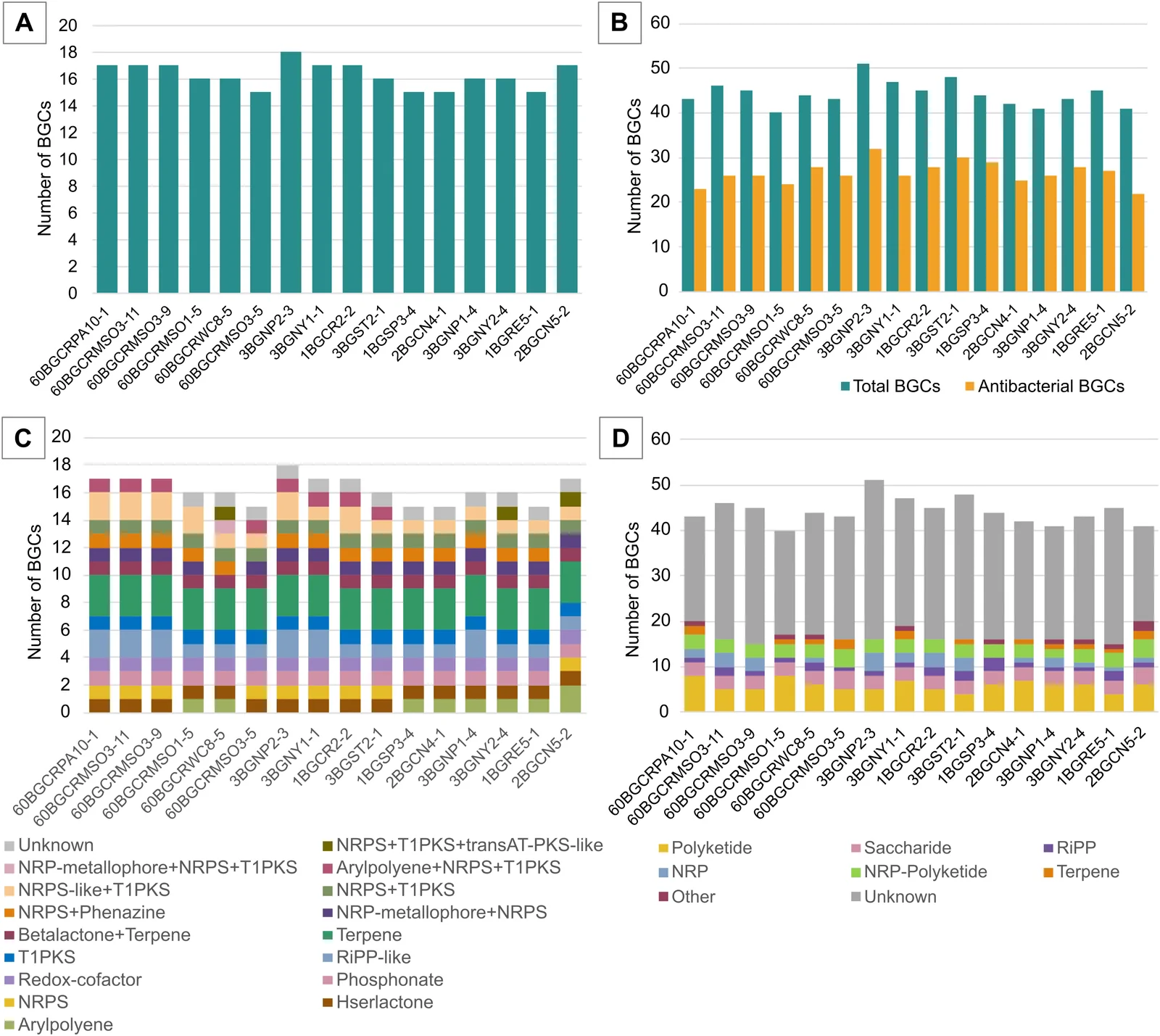
Genome mining of BGCs in 16 *B. glumae* isolates from Thailand. (A) Number of BGCs identified by antiSMASH. (B) Number of total and antibacterial-associated BGCs predicted by DeepBGC. (C) Composition of antiSMASH-identified BGC classes. (D) Composition of the DeepBGC-predicted product classes. The BGC classes predicted by DeepBGC but not assigned to a known product class were categorized as unknowns.

The antiSMASH analysis identified diverse classes of BGCs across the isolate collection (Fig 1C, S2 Table). The 16 *B. glumae* isolates showed high conservation among several BGC classes, including terpene, type I polyketide synthase (T1PKS), ribosomally synthesized and post-translationally modified peptide (RiPP)-like, nonribosomal peptide synthetases (NRPS)-like+T1PKS, homoserine lactone (hserlactone), phosphonate, redox-cofactor, and betalactone+terpene clusters. Terpene-associated BGCs were particularly abundant, with three clusters identified in every genome. Most isolates also contained two RiPP-like and two NRPS-like+T1PKS clusters, while NRP-metallophore+NRPS and NRPS+phenazine clusters were detected in 15 of the 16 isolates.

DeepBGC predicted that 22–32 BGCs per isolate were associated with antibacterial activity (Fig 1B, S3 Table). These antibacterial-associated BGCs accounted for approximately half of all the predicted BGCs in most isolates, indicating that the isolates had genomic capacity for the production of metabolites with potential antimicrobial activity. The predicted BGCs were assigned to multiple product classes, including polyketides, nonribosomal peptides (NRPs), NRP–polyketide hybrids, saccharides, RiPPs, terpenes, and other categories (Fig 1D, S3 Table). The polyketide-, NRP–polyketide-, RiPP-, and saccharide-associated clusters were among the most abundant product classes. Because a large proportion (>50%) of the predicted BGCs could not be assigned to a known product class, they were categorized as unknowns, indicating that a large fraction of the biosynthetic potential encoded within these genomes remains uncharacterized (Fig 1D, S3 Table).

### Evaluation of the antibacterial activity of *B. glumae* isolates

The antibacterial activity of the 16 *B. glumae* isolates was evaluated using an overlay assay on LB agar plates. All isolates exhibited the same antibacterial spectrum, showing inhibitory activity against *A. baumannii* ATCC 19606, *E. coli* ATCC 25922, and *S. aureus* ATCC 29213, but no inhibition against *K. pneumoniae* ATCC 700603 or *P. aeruginosa* ATCC 27853. Clear-zone diameters were measured in three independent experiments. Mean values with error bars representing standard deviations (SD) are presented in Fig 2, and the corresponding numerical values are provided in S4 Table. Based on the average inhibition zone diameter for *A. baumannii*, *E. coli*, and *S. aureus*, four isolates (60BGCRMSO1–5, 60BGCRWC8–5, 1BGCR2–2, and 1BGSP3–4) exhibiting the strongest overall antibacterial activity were selected for further characterization using the agar plug assay. The observation of clear inhibition zones surrounding the agar plugs when using the susceptible indicator strains demonstrated that antibacterial compounds produced by these isolates diffused into the surrounding agar medium. Representative results from the overlay and agar plug diffusion assays are shown in Fig 3. These four isolates were selected for subsequent metabolomic analysis.

**Fig 2:**
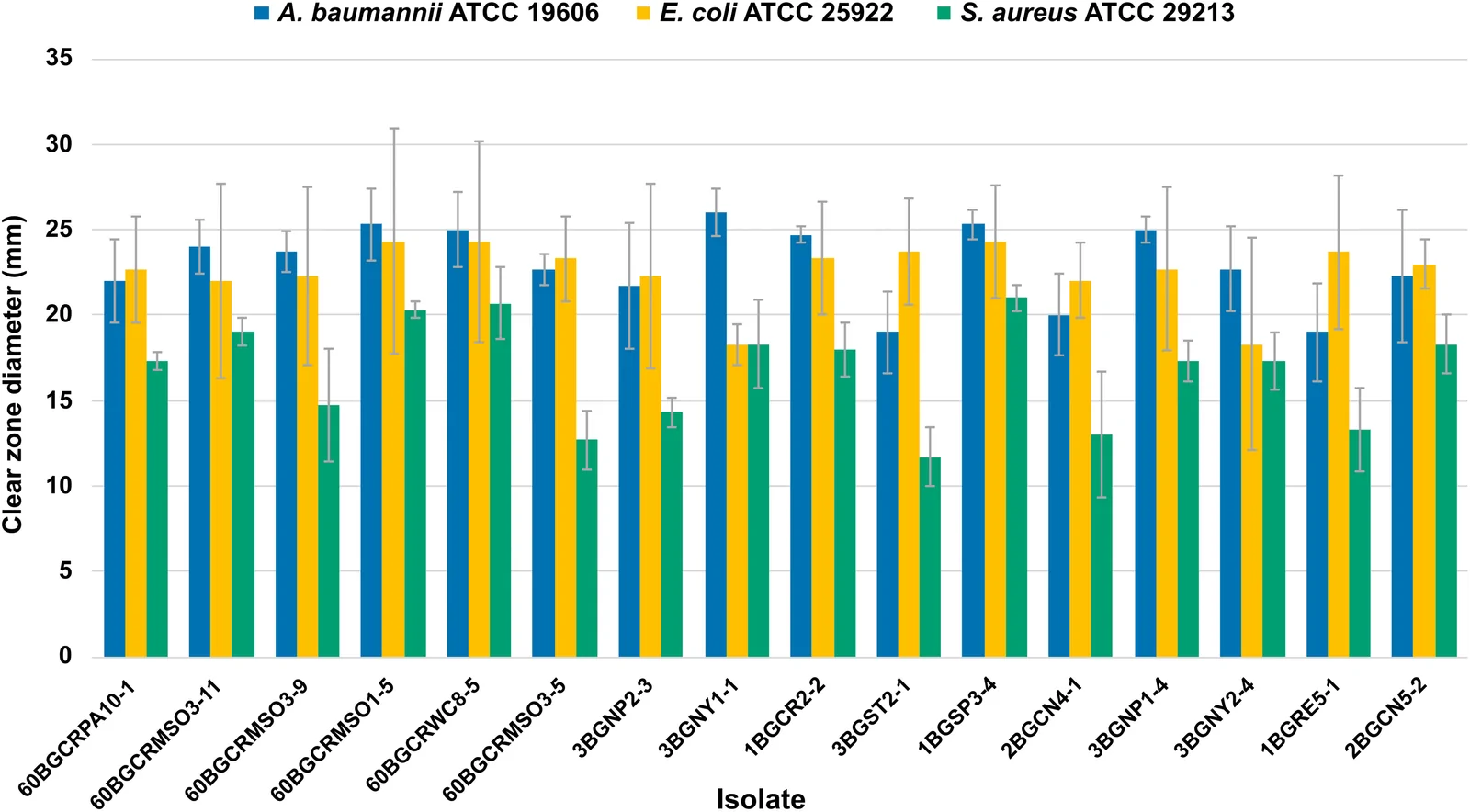
Antibacterial activity of *Burkholderia glumae* isolates from Thailand. Inhibition zone diameters against *Acinetobacter baumannii* ATCC 19606, *Escherichia coli* ATCC 25922, and *Staphylococcus aureus* ATCC 29213 were measured using soft-agar overlay assays on LB agar. Bars represent mean inhibition zone diameters (mm), and error bars indicate standard deviations from three independent experiments.

**Fig 3:**
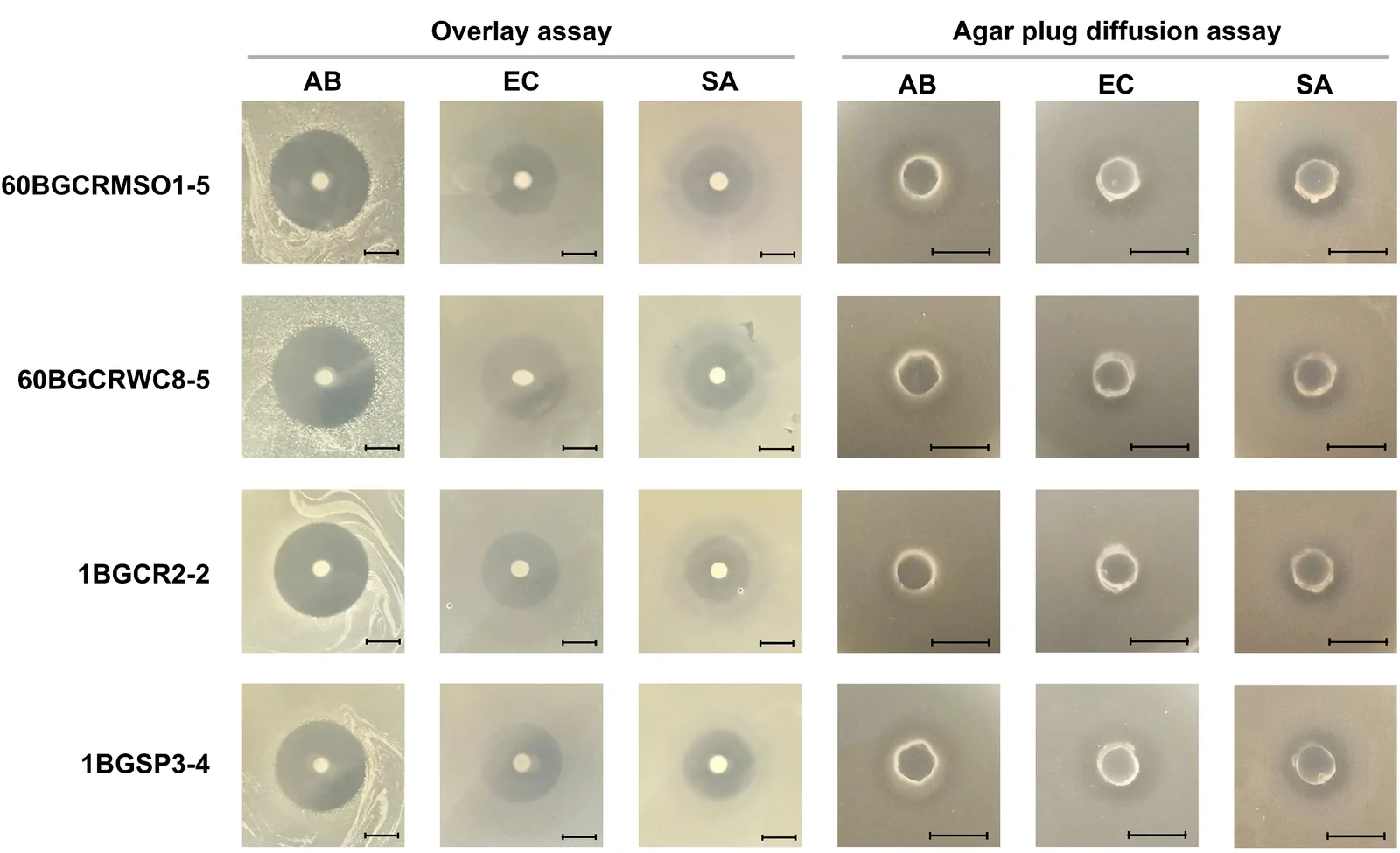
Antibacterial activity of selected *Burkholderia glumae* isolates. Representative images of overlay assays and agar plug diffusion assays performed against *Acinetobacter baumannii* ATCC 19606 (AB), *Escherichia coli* ATCC 25922 (EC), and *Staphylococcus aureus* ATCC 29213 (SA). Scale bars = 10 mm.

### Metabolomic analysis of selected *B. glumae* isolates

Untargeted LC–MS analysis was performed on extracts from three biological replicates of LB agar cultures of each of the four selected *B. glumae* isolates exhibiting the strongest antibacterial activity. The resulting metabolomic profiles were compared with those of extracts from uninoculated LB agar plates used as medium controls. PCA of QC samples demonstrated tight clustering of the QC injections, indicating good analytical stability and reproducibility throughout the LC–MS analysis (S1 Fig). PCA of the *B. glumae* isolates and uninoculated LB agar controls revealed clear separation between isolate-derived and control metabolite profiles (Fig 4A). Replicate samples clustered closely within each group, further supporting the reproducibility of the metabolomic data. Consistent with the PCA results, hierarchical clustering analysis showed that replicates from the same isolate grouped together and were distinct from control samples (Fig 4B). The metabolomic profiles of isolates 1BGCR2−2 and 1BGSP3−4 were highly similar, whereas 60BGCRMSO1−5 and 60BGCRWC8−5 formed a second closely related cluster.

**Fig 4:**
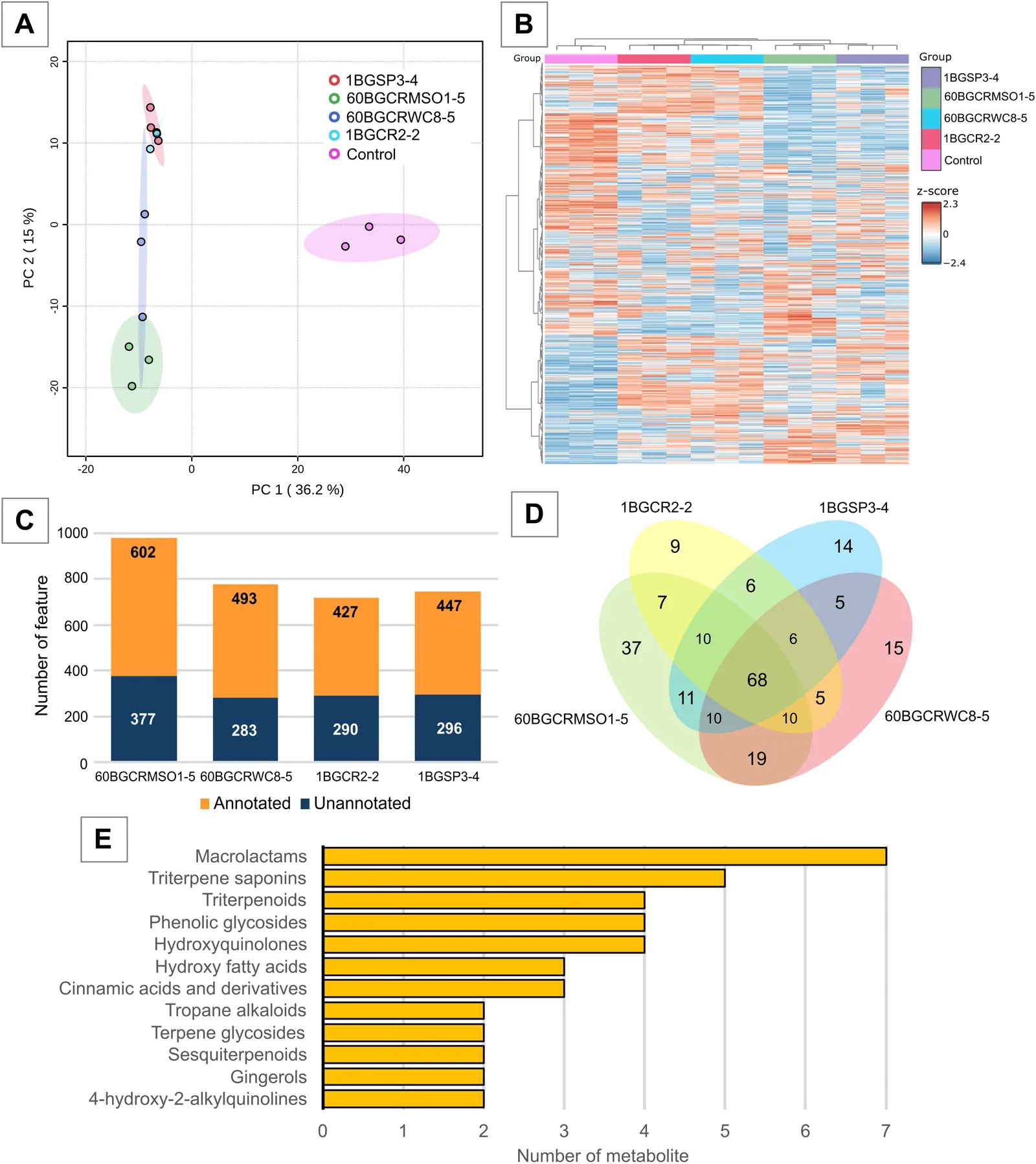
Metabolomic profiling of selected *Burkholderia glumae* isolates. (A) PCA score plot showing the metabolomic profiles of four selected *B. glumae* isolates and uninoculated LB agar controls. Each point represents an individual biological replicate. (B) Hierarchical clustering heatmap of z-score-normalized metabolite abundance profiles across the four *B. glumae* isolates and uninoculated Luria-Bertani (LB) agar controls. (C) Number of metabolites showing significantly increased abundance (fold change > 2, *p* < 0.05) relative to uninoculated LB agar, classified according to annotation status. (D) Four-set Venn diagram showing putatively annotated antimicrobial-related metabolites shared among the four *B. glumae* isolates. (E) Distribution of chemical classes among the 68 putatively annotated antimicrobial-related metabolites shared by all four *B. glumae* isolates. Only classes represented by two or more metabolites are shown.

Metabolites were putatively annotated based on MS/MS spectral matching against the MS-DIAL ESI (+/−) database. Relative to uninoculated LB agar, 717–979 metabolites exhibited significantly increased abundance (fold change > 2, *p* < 0.05) across the four isolates (Fig 4C). Approximately 60–65% of these metabolites were successfully annotated, whereas 35–40% remained unannotated, reflecting limitations of current spectral databases and the potential presence of previously uncharacterized compounds (Fig 4C, S5 Table).

Metabolites potentially associated with antibacterial activity were identified by further examination of the annotated metabolites belonging to chemical classes with previously reported antimicrobial properties. A total of 68 putatively annotated antimicrobial-related metabolites shared among all four isolates were assigned to 40 chemical classes (Fig 4D, S6 Table). Figure 4E highlights the classes represented by at least two metabolites. Macrolactams were the most frequently detected class (n = 7), followed by triterpene saponins (n = 5), and triterpenoids, phenolic glycosides, and hydroxyquinolones (n = 4 each).

Among the 68 shared putatively antimicrobial metabolites, 15 metabolites exhibited greater than five-fold enrichment relative to uninoculated LB agar (Table 1). These highly enriched metabolites belonged primarily to the triterpene saponin, cyclic depsipeptide, macrolactam, and triterpenoid classes, with fold changes ranging from 5.2 to 49.3.

**Table 1:** Putatively annotated metabolites with significantly higher abundance (fold change > 5, p < 0.05) compared to uninoculated Luria-Bertani agar, assigned to chemical classes with previously reported antimicrobial activity.

No	Average m/z	Average RT (min)	Adduct type	Mass error (ppm)	Putative metabolite class	Average fold change			
						60BGCRMSO1–5	60BGCRWC8−5	1BGCR2−2	1BGSP3–4
1	1116.58	8.149	[M+H]+	12.47	Triterpene saponins	22.14	26.74	39.87	49.29
2	745.059	8.152	[M+Na]+	−17.86	Cyclic depsipeptides	19.22	35.82	24.5	28.9
3	1127.58	8.148	[M+Na]+	−13.21	Triterpene saponins	14.74	20.07	21.43	23.99
4	585.36	3.661	[M+H]+	−0.51	Macrolactams	15.53	16.49	25.69	12.99
5	681.394	5.775	[M-H]–	−11.64	Triterpenoids	14.78	17.09	15.1	21.69
6	455.25	3.877	[M-H]–	−14.01	Sesquiterpenoids	11.43	11.58	14.98	12.7
7	683.409	5.786	[M+H]+	−13.4	Triterpene saponins	10.01	12.83	11.53	10.74
8	573.331	8.17	[M+H]+	−8.62	Macrolactams	7.64	15.17	8.1	11.04
9	615.009	8.154	[M+Na]+	−14.29	Macrolides and analogues	7.9	8.28	7.59	7.99
10	225.004	1.568	[M-H]–	12.93	Organosulfonic acids	9.89	8.87	7.59	5.79
11	373.256	2.996	[M+H]+	17.74	Gingerols	5.57	12.3	8.23	5.7
12	288.155	2.439	[M-H]–	18.84	Tropane alkaloids	7.12	7.28	7.28	7.84
13	373.257	2.699	[M+H]+	15.94	Gingerols	6.69	6.34	7.9	7.55
14	359.24	2.376	[M+H]+	−20.04	Beta carbolines	6.8	8.23	6.49	5.21
15	294.183	2.467	[M+2H]2+	−24.71	Macrolactams	5.53	5.92	5.28	5.18

A comparison of genome-mining predictions and metabolomic findings is presented in Table 2. antiSMASH identified terpene-, polyketide-, and NRPS-associated regions in all four selected isolates, alongside metabolomic features putatively assigned to terpene-related, macrolactam, and peptide-related classes. Targeted BLASTn analysis also detected a region corresponding to the toxoflavin biosynthesis-associated locus in all four isolates with >99% nucleotide identity and query coverage, although toxoflavin was not detected. The biosynthetic origins of the alkylquinoline-related and remaining metabolomic features were unresolved. These comparisons summarize potential class-level correspondences without establishing direct links between individual BGCs and metabolites.

**Table 2:** Summary of genome-mining predictions and metabolomic findings in the four selected *Burkholderia glumae* isolates.

Biosynthetic category	Genome-mining findings	Metabolomic findings
Terpene-associated biosynthesis	antiSMASH identified three terpene regions and one betalactone–terpene region in each isolate. DeepBGC predicted terpene-associated clusters in two of the four isolates.	Features putatively classified as triterpene saponins (n = 5), triterpenoids (n = 4), sesquiterpenoids (n = 2), terpene glycosides (n = 2), diterpenoids (n = 1), and terpene lactones(n = 1).
Polyketide and hybrid NRPS–PKS biosynthesis	antiSMASH identified one T1PKS region and 2–4 hybrid NRPS–PKS regions per isolate. DeepBGC predicted 5–8 polyketide and three NRP–polyketide clusters per isolate.	Features putatively classified as macrolactams (n = 7) and macrolides and analogues (n = 1).
Nonribosomal peptide-associated biosynthesis	antiSMASH identified 4–7 NRPS-or NRPS-like-containing regionsper isolate. DeepBGC predicted three NRP–polyketide clusters per isolate.	Features putatively classified as cyclic depsipeptides (n = 1) and oligopeptides (n = 1).
Alkylquinoline-associated metabolites	The biosynthetic origin remains unresolved.	Features putatively classified as hydroxyquinolones (n = 4) and4-hydroxy-2-alkylquinolines(n = 2).
Toxoflavin biosynthesis	Targeted BLASTn analysis identified the toxoflavin biosynthesis-associated locus in all four selected isolates, with >99% nucleotide identity and query coverage.	Toxoflavin was not detected under the conditions examined.
Other predictions and metabolomic features	Additional predictions included phosphonate, redox-cofactor, homoserine lactone, RiPP-like, and other or unclassified regions.	The remaining 36 features were assigned to other chemical classes, with no specific correspondence established with the predicted BGCs.

## Discussion

Our goal in this study was to integrate genome mining, antibacterial screening, and untargeted metabolomics to conduct a multifaceted investigation of the antimicrobial potential of *B. glumae* isolates collected from Thailand. To do this, we conducted genome mining, which successfully identified multiple BGCs across all isolates, with both antiSMASH and DeepBGC predicting diverse classes of specialized metabolite biosynthetic pathways. Notably, approximately half of the DeepBGC-predicted clusters were classified as antibacterial-associated, consistent with previous reports of antimicrobial activity in *B. glumae* and providing genomic evidence for its capacity to produce antimicrobial compounds [20,21].

The antibacterial screening results demonstrated that all isolates exhibited activity against *A. baumannii*, *E. coli*, and *S. aureus*, in agreement with the genome mining predictions and indicating that at least part of the encoded biosynthetic potential is expressed under the cultivation conditions used in this study. The consistent inhibition spectrum observed across all isolates is also consistent with the highly conserved BGC composition identified by antiSMASH and DeepBGC. Collectively, these findings suggest that antimicrobial metabolite production may represent a common trait among the examined *B. glumae* isolates. Previous studies that have evaluated *B. glumae* isolates from Japan, the Philippines, the United States, and Vietnam also reported antibacterial activity against *A. baumannii* and *S. aureus*; however, only 10 of the 15 isolates examined showed a capacity to inhibit *S. aureus* and only 2 of the 15 inhibited *A. baumannii* in overlay assays [21]. In contrast, all 16 isolates from Thailand examined in the present study inhibited both of these pathogens.

This difference between studies may reflect variations in the isolate collections. Alternatively, differences in cultivation conditions may have influenced metabolite production and antibacterial activity. The previous study cultivated *B. glumae* on basal salts medium supplemented with glycerol (BSM-G) agar at 28 °C for four days [21], whereas the isolates examined in the present study were incubated on LB agar at 37 °C for 24 h. Given that specialized metabolite production is often influenced by nutrient availability, temperature, and growth phase, differences in cultivation conditions may have altered the expression of biosynthetic pathways and the resulting antibacterial metabolite profiles [35].

In the present study, untargeted LC–MS metabolomic analysis identified 68 putatively annotated antimicrobial-related metabolites that were shared among the four most active isolates and that were significantly enriched compared with uninoculated LB agar. These metabolites were assigned to diverse classes, including macrolactams, triterpene saponins, triterpenoids, and cyclic depsipeptides, which have previously been associated with antimicrobial activity [36–39]. Although these metabolite annotations remain tentative and require structural confirmation, they identify a set of candidate metabolites that may contribute to the observed antibacterial activity. Furthermore, the metabolomic findings presented here are broadly consistent with the genome mining results, which identified multiple BGCs associated with terpenes, polyketides, and hybrid NRPS–polyketides [37,40–42].

Among the antimicrobial-related metabolites shared by the isolates from Thailand, macrolactams and triterpene saponins were of particular interest. Macrolactams represented the most frequently detected metabolite class, with several metabolites exhibiting greater than five-fold enrichment (up to 25.7-fold) relative to uninoculated LB agar, whereas triterpene saponins exhibited the highest levels of enrichment, with fold changes as high as 49.3-fold. The detection of macrolactams is compatible with the presence of BGCs associated with PKS and PKS–NRPS, as predicted by antiSMASH and DeepBGC [37,40], whereas the occurrence of triterpene-related metabolites is consistent with the conserved BGCs associated with terpenes identified across all isolates [41]. Although this study did not establish direct links between specific metabolites and biosynthetic gene clusters, the consistency between the genome mining data and metabolomic analyses supports the prediction that *B. glumae* has the capacity to produce structurally diverse antibacterial metabolites.

A previous investigation of antimicrobial-active *B. glumae* isolates reported the detection of toxoflavin, reumycin, bactobolin A and two potentially novel antibacterial compounds [21]. However, none of these metabolites were detected in the present study. Targeted BLASTn analysis identified a region corresponding to the toxoflavin biosynthesis-associated locus in all four selected isolates. Nevertheless, sequence conservation alone does not establish functional expression or metabolite production. Toxoflavin biosynthesis and transport in *B. glumae* are regulated by ToxJ, whose expression is activated by quorum sensing, and the LysR-type regulator ToxR, which requires toxoflavin as a coinducer [43]. Differences in cultivation conditions may have influenced the metabolite profiles observed across studies. The previous study cultivated isolates on a nutrient-limited medium at 28 °C for four days [21], whereas the present study used LB agar at 37 °C for 24 h. Toxoflavin production has also been observed in *B. glumae* BGR1 grown on LB agar at 30 °C for 48 h [44], suggesting that nutrient-rich conditions do not inherently suppress its synthesis. These observations indicate that variables such as incubation temperature and duration may account for inter-study variations, though their direct influence was not investigated in the current study. Reumycin serves as a biosynthetic precursor of toxoflavin [30], suggesting that variation in pathway activity may influence the abundance and detectability of both compounds. Although bactobolin A was detected in the previous study [21], its biosynthetic basis and the factors regulating its production in *B. glumae* remain unclear.

The identification of numerous predicted BGCs, together with antibacterial activity and diverse metabolite profiles, highlights the potential of *B. glumae* isolates from Thailand as a source of antimicrobial metabolites. Importantly, many of the DeepBGC-predicted clusters could not be assigned to known product classes, suggesting that the biosynthetic potential of *B. glumae* is still incompletely characterized. Likewise, many metabolomic features remained unannotated, highlighting the current limitations in metabolite databases and indicating the potential presence of previously uncharacterized metabolites.

Several limitations of the present study should be considered. One was that the metabolite annotations were based primarily on mass spectral matching and chemical class prediction; therefore, they remain putative. Furthermore, the metabolites responsible for antibacterial activity were neither experimentally isolated nor validated. Future studies should incorporate bioassay-guided fractionation, purification of active compounds, and structural characterization using nuclear magnetic resonance spectroscopy to verify the identities and antimicrobial activities of metabolites produced by the *B. glumae* isolates from Thailand. Further integration of genome mining with metabolite networking and genome–metabolite association analyses may facilitate the linkage of specific BGCs to their corresponding metabolites. These approaches will be necessary to identify the compounds responsible for antibacterial activity and to evaluate their potential for antimicrobial development.

## Conclusion

This study used an integrative approach that included genome mining, antibacterial screening, and untargeted metabolomic profiling to investigate the antimicrobial potential of *B. glumae* isolates from Thailand. Genome mining revealed diverse BGCs related to antimicrobial metabolites, including terpene-, polyketide-, and NRPS-associated pathways. Phenotypic assays demonstrated consistent antibacterial activity against *A. baumannii*, *E. coli*, and *S. aureus*. Metabolomic analysis identified multiple, significantly enriched, putatively antimicrobial-related metabolites shared among the most active isolates, including macrolactams and triterpene saponins. The concordance between genome mining, antibacterial activity, and metabolomic profiles supports the capacity of *B. glumae* to produce structurally diverse antimicrobial metabolites. Together, these findings highlight *B. glumae* isolates from Thailand as a promising source of candidate metabolites for future antimicrobial discovery efforts.

## Supporting information

S1 Fig Principal component analysis score plots of untargeted LC–MS data from four selected *Burkholderia glumae* isolates, uninoculated LB agar controls, and quality control samples. (PDF)

S1 Table Processed LC-MS/MS feature table exported from MS-DIAL, including feature IDs, retention times, m/z values, putative metabolite annotations, identification scores, and peak intensities for all samples. (XLSX)

S2 Table antiSMASH-predicted biosynthetic gene clusters identified in 16 *Burkholderia glumae* genomes. (XLSX)

S3 Table DeepBGC-predicted biosynthetic gene clusters and antibacterial-associated biosynthetic gene clusters identified in 16 *Burkholderia glumae* genomes. (XLSX)

S4 Table Inhibition zone diameters produced by Burkholderia glumae isolates against selected human bacterial pathogens, as determined by overlay assays on LB agar. (PDF)

S5 Table Metabolites showing significantly increased abundance in four selected *Burkholderia glumae* isolates compared with uninoculated LB agar controls, identified by untargeted LC–MS/MS analysis. (XLSX)

S6 Table Putatively annotated antimicrobial-related metabolites shared among the four selected *Burkholderia glumae* isolates. (XLSX)
