# Supplementary material for: Genome mining, antibacterial activity, and metabolomic profiling reveal the biosynthetic potential of Burkholderia glumae isolates from Thailand with activity against clinically important bacterial pathogens
Source: PLoS One. 2026 Oct 30;21(10):e0361711. doi: 10.1371/journal.pone.0361711 (PMC13722174; doi:10.1371/journal.pone.0361711)
Supplement: Supplementary file 1 [file pone.0361711.s001.pdf]

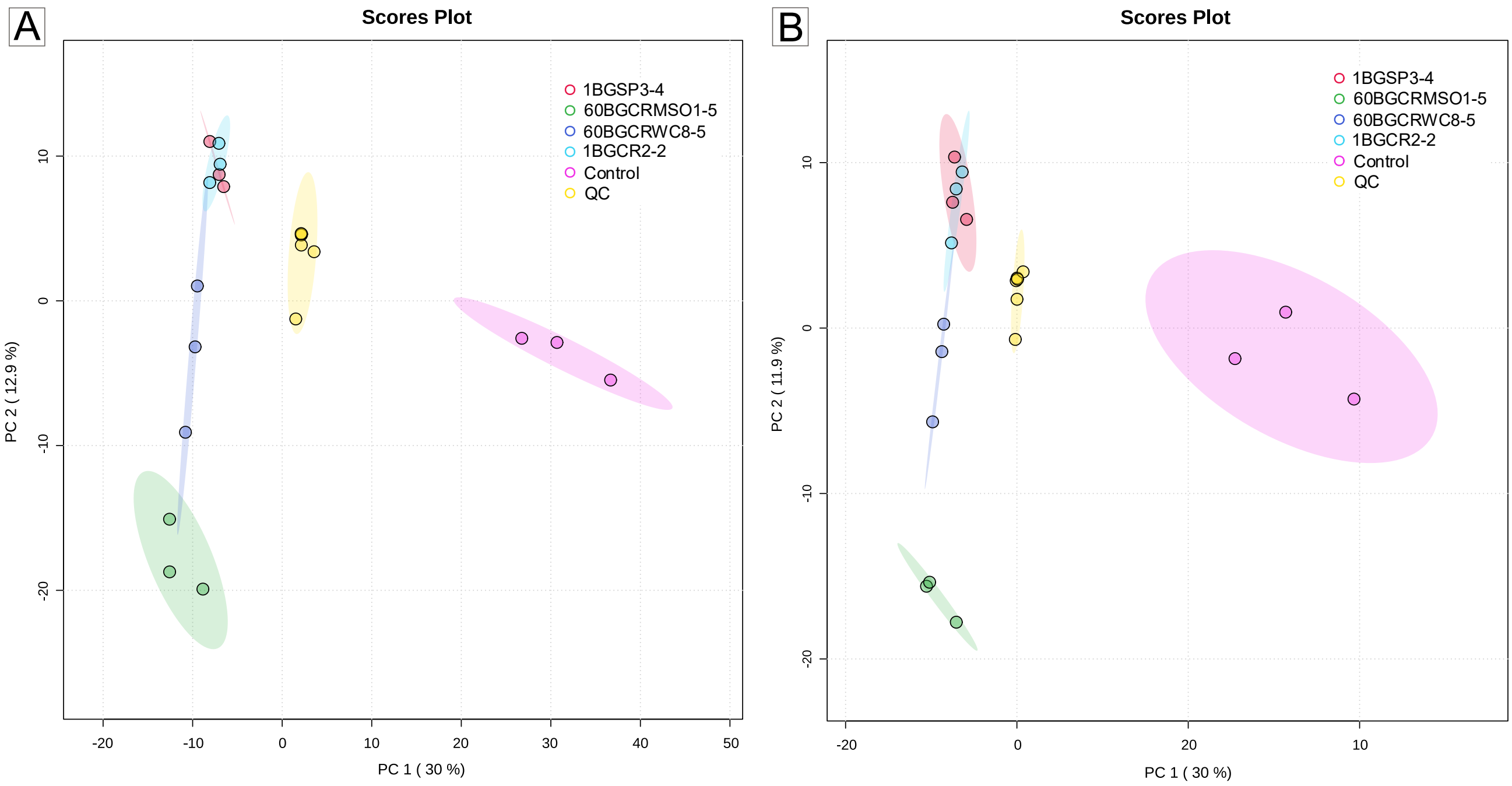

Fig S1. Principal component analysis (PCA) score plots of untargeted LC–MS data from four selected *Burkholderia glumae* isolates, uninoculated LB agar controls, and quality control (QC) samples. Three biological replicates were analyzed for each isolate and control. (A) Positive ionization mode. (B) Negative ionization mode.
