# Supplementary material for: Genome mining, antibacterial activity, and metabolomic profiling reveal the biosynthetic potential of Burkholderia glumae isolates from Thailand with activity against clinically important bacterial pathogens
Source: PLoS One. 2026 Oct 30;21(10):e0361711. doi: 10.1371/journal.pone.0361711 (PMC13722174; doi:10.1371/journal.pone.0361711)
Supplement: Supplementary file 2 [file pone.0361711.s005.pdf]

Table S4. Inhibition zone diameters produced by *Burkholderia glumae* isolates against selected human bacterial pathogens, as determined by overlay assays on LB agar.

Isolate	Clear zone diameter (mm)*		
	AB	EC	SA
60BGCRPA10-1	22.0 ± 2.4	22.7 ± 3.1	17.3 ± 0.5
60BGCRMSO3-11	24.0 ± 1.6	22.0 ± 5.7	19.0 ± 0.8
60BGCRMSO3-9	23.7 ± 1.2	22.3 ± 5.2	14.7 ± 3.3
60BGCRMSO1-5	25.3 ± 2.1	24.3 ± 6.6	20.3 ± 0.5
60BGCRWC8-5	25.0 ± 2.2	24.3 ± 5.9	20.7 ± 2.1
60BGCRMSO3-5	22.7 ± 0.9	23.3 ± 2.5	12.7 ± 1.7
3BGNP2-3	21.7 ± 3.7	22.3 ± 5.4	14.3 ± 0.9
3BGNY1-1	26.0 ± 1.4	18.3 ± 1.2	18.3 ± 2.6
1BGCR2-2	24.7 ± 0.5	23.3 ± 3.3	18.0 ± 1.6
3BGST2-1	19.0 ± 2.4	23.7 ± 3.1	11.7 ± 1.7
1BGSP3-4	25.3 ± 0.9	24.3 ± 3.3	21.0 ± 0.8
2BGCN4-1	20.0 ± 2.4	22.0 ± 2.2	13.0 ± 3.7
3BGNP1-4	25.0 ± 0.8	22.7 ± 4.8	17.3 ± 1.2
3BGNY2-4	22.7 ± 2.5	18.3 ± 6.2	17.3 ± 1.7
1BGRE5-1	19.0 ± 2.9	23.7 ± 4.5	13.3 ± 2.4
2BGCN5-2	22.3 ± 3.9	23.0 ± 1.4	18.3 ± 1.7

*Clear zone diameters are presented as mean ± SD from three independent experimental replicates (n = 3). AB, *Acinetobacter baumannii* ATCC 19606; EC, *Escherichia coli* ATCC 25922; SA, *Staphylococcus aureus* ATCC 29213.
