# Supplementary material for: Genome mining, antibacterial activity, and metabolomic profiling reveal the biosynthetic potential of Burkholderia glumae isolates from Thailand with activity against clinically important bacterial pathogens
Source: PLoS One. 2026 Oct 30;21(10):e0361711. doi: 10.1371/journal.pone.0361711 (PMC13722174; doi:10.1371/journal.pone.0361711)
Supplement: Supplementary file 3 [file pone.0361711.s009.pdf]

Response to the reviewer comments

Reviewer #1:

This is an interesting paper on the genome mining, antimicrobial activity, and metabolite profiling of Burkholderia glumae strains from Thailand. The authors have done a good job in preparing this manuscript, and I recommend publication after minor to moderate revision.

Specific Comments

Line 44: Please clarify what is meant by "uninoculated controls".

Thank you for this comment. We have clarified that the uninoculated controls consisted of LB agar plates without bacterial inoculation that were incubated under the same conditions and processed using the same metabolite extraction and LC–MS/MS procedures as the inoculated samples.

We have revised the Abstract accordingly.

The original text:

"Macrolactams represented the most frequently detected metabolite class, whereas triterpene saponins exhibited the highest enrichment relative to uninoculated controls."

Has been revised to:

"Macrolactams represented the most frequently detected metabolite class, whereas triterpene saponins exhibited the highest enrichment relative to control LB agar without bacterial inoculation."

We have also added the following clarification to the LC–MS/MS-based metabolite profiling subsection of the Materials and Methods.

"Uninoculated LB agar plates prepared without bacterial inoculation and incubated under the same conditions were used as medium controls and subjected to the same collection, extraction, and LC–MS/MS procedures as the inoculated samples."

Line 60: This statement could be updated to include Escherichia coli, and the acronym should be extended to ESKAPEE pathogens, as E. coli was also included in the antimicrobial screening.

Thank you for this suggestion. We have expanded the acronym from ESKAPE to ESKAPEE and added *Escherichia coli* to the list of pathogens, as this species was included in our antimicrobial screening. We have also replaced the original reference with one that specifically supports the expanded ESKAPEE classification.

The revised text reads:

"Particularly concerning are the ESKAPEE pathogens (*Enterococcus faecium, Staphylococcus aureus, Klebsiella pneumoniae, Acinetobacter baumannii, Pseudomonas aeruginosa, Enterobacter* spp. and *Escherichia coli*), which account for a significant proportion of multidrug-resistant healthcare-associated infections worldwide [3]."

Line 70: Please replace "secondary metabolites" with "specialised metabolites" throughout the manuscript. The term "secondary metabolites" is considered outdated, as it incorrectly implies that these compounds are of secondary importance, whereas they are highly specialised molecules with important ecological functions.

Thank you for this important suggestion. We agree that "specialized metabolites" more accurately reflects the ecological and biological functions of these compounds. We have therefore replaced "secondary metabolites" with "specialized metabolites" and made corresponding changes to related phrases throughout the manuscript, including the abstract, keywords, introduction, results, and discussion.

Lines 110-111: This statement requires an appropriate reference.

Thank you for pointing this out. We have added appropriate references supporting the use of genome mining and metabolomics as complementary tools for natural product discovery.

Line 117: The two cited references in this sentence should be removed as they are not specific to B. glumae and do not directly are not directly related to this study's objectives.

Thank you for this comment. We agree that the cited references are not specific to *B. glumae* and are unnecessary in a statement describing the objectives of the present study. We have therefore removed both references from this sentence.

Line 119: Change BGSs to BGCs

Thank you for identifying this typographical error. We have corrected "BGSs" to "BGCs."

Line 123: Please revise to the more formal wording "B. glumae isolates from Thailand". The manuscript title should also be amended, if appropriate, to reflect this more formal taxonomic description. Please apply this change consistently throughout the manuscript.

Thank you for this suggestion. We have replaced "Thai *B. glumae* isolates" with the more formal wording "*B. glumae* isolates from Thailand." We have also amended the manuscript title and applied this change consistently throughout the manuscript.

The original title:

"Genome mining, antibacterial activity, and metabolomic profiling reveal the biosynthetic potential of Thai *Burkholderia glumae* isolates with activity against clinically important bacterial pathogens"

Has been revised to:

"Genome mining, antibacterial activity, and metabolomic profiling reveal the biosynthetic potential of *Burkholderia glumae* isolates from Thailand with activity against clinically important bacterial pathogens"

Line 135: What medium were the bacterial stocks prepared in prior to storage with 20% glycerol? Please provide the composition/name of the medium and any relevant details.

Thank you for this comment. We have clarified the procedure used to prepare the bacterial stocks. Briefly, the bacterial isolates were cultured on LB agar, and colonies were collected directly from the agar surface, suspended in sterile 20% (v/v) glycerol solution, and stored at -80 °C until use. The revised information has been added to the Materials and Methods section.

The original text:

"Bacterial stock cultures were stored at -80 °C in 20% glycerol until use."

Has been revised to:

"Before storage, each bacterial isolate was cultured on Luria–Bertani (LB) agar, Miller (Difco, Becton, Dickinson and Company, USA). Colonies were collected directly from the agar surface, suspended in sterile 20% (v/v) glycerol solution, and stored at -80 °C until use."

Line 147: Please specify whether the LB agar used was the Lennox or Miller formulation and provide the manufacturer's details. In addition, LB should not be defined as Luria-Bertani medium unless this is the designation used by the manufacturer, as the acronym LB does not universally stand for Luria-Bertani.

Thank you for this comment. We have clarified that the medium used was the Miller formulation and added the manufacturer's details. Because "Luria-Bertani" appears in the manufacturer's official product designation, LB is defined accordingly at its first occurrence as "Luria–Bertani (LB) agar, Miller (Difco, Becton, Dickinson and Company, USA)."

Line 157: Please confirm whether the 8 mL overlay was sterile molten soft agar or soft LB agar and provide the medium composition.

Thank you for this comment. We confirm that the overlay consisted of sterile molten soft agar rather than soft LB agar. We have revised the Materials and Methods section to specify that the soft agar contained 0.8% (w/v) bacteriological agar in deionized water.

The original text:

"For the overlay assay, 80 μL of each indicator culture was mixed with 8 mL of sterile molten soft agar (0.8% agar) maintained at 42 °C, gently mixed, and poured over the agar plates containing the pre-grown *B. glumae* spots."

Has been revised to:

For the overlay assay, 80 μL of each indicator culture was added to 8 mL of sterile molten soft agar (0.8% [w/v] bacteriological agar in deionized water) maintained at 42 °C, gently mixed, and poured over the agar plates containing the pre-grown *B. glumae* spots.

Line 167: Similarly, please clarify if this is soft agar or soft LB agar.

Thank you for this comment. We confirm that the same sterile molten soft agar described in the preceding subsection (0.8% [w/v] bacteriological agar in deionized water) was used for the agar plug assay, rather than soft LB agar. To avoid unnecessary repetition while

ensuring clarity, we have revised the text to state that the molten soft agar was "prepared as described above."

The revised text reads:

"An aliquot (80 µL) of each culture was mixed with 8 mL of sterile molten soft agar prepared as described above, maintained at 42 °C and overlaid onto LB agar plates."

Line 233: This is the first mention of the controls in the Materials and Methods section. Please provide a description of the control treatments, including their composition and purpose, at an earlier point in the Methods to ensure the experimental design is clearly explained.

Thank you for this comment. We have added a description of the control treatment at an earlier point in the LC–MS/MS-based metabolite profiling subsection.

"Uninoculated LB agar plates prepared without bacterial inoculation and incubated under the same conditions were used as medium controls and subjected to the same collection, extraction, and LC–MS/MS procedures as the inoculated samples."

Line 243: Change BCG to BGC

Thank you for identifying this typographical error. We have corrected "BCG" to "BGC" in the revised manuscript.

Line 366: Please rephrase this statement, as antimicrobial activity in B. glumae has already been reported in the literature and is therefore not merely a prediction.

Thank you for this comment. We agree that antimicrobial activity in *B. glumae* has already been demonstrated previously and should not be described merely as a prediction. We have revised the statement to clarify that our genome-mining findings are consistent with previous reports of antimicrobial activity and provide additional genomic evidence for the capacity of *B. glumae* to produce antimicrobial compounds.

The original text:

"Notably, approximately half of the DeepBGC-predicted clusters were classified as antibacterial-associated, supporting the prediction that B. glumae possesses genomic capacity for the production of antimicrobial compounds."

Has been revised to:

"Notably, approximately half of the DeepBGC-predicted clusters were classified as antibacterial-associated, consistent with previous reports of antimicrobial activity in *B. glumae* and providing genomic evidence for its capacity to produce antimicrobial compounds [20,21]."

Line 378: Change to ....all 16 of the isolates from Thailand....

Thank you for this suggestion. We have revised the text to "all 16 isolates from Thailand" for clarity and conciseness.

Line 378: Did the authors analyse the B. glumae genomes reported by Depoorter et al. or other publicly available genomes to determine whether differences exist in biosynthetic gene cluster (BGC) content compared with the isolates examined in this study?

Thank you for raising this important point. We did not perform a comparative BGC analysis with the isolates examined by Depoorter et al. because their genome sequences were not reported or publicly available. Although genomes of other *B. glumae* isolates are publicly available, they generally lack corresponding antibacterial activity data and information on cultivation conditions. Consequently, while comparison with these genomes could provide information on predicted BGC diversity, interpretation of any differences in relation to antibacterial activity or metabolite production would be limited. We have therefore retained the present analysis, which focuses on the genomically and phenotypically characterized isolates from Thailand included in this study.

Lines 382-384: The citation placement should be revised. Reference (20) should be cited immediately after "for four days" rather than at the end of the sentence. Its current position implies that the citation supports the statement regarding the isolates from Thailand, rather than on BSMG.

Thank you for this comment. We have moved the citation to immediately after “for four days,” as suggested, to clarify that it refers to the cultivation conditions used in the previous study.

Line 396, 403, 405: Change BCGs to BGCs

Thank you for identifying these typographical errors. We have corrected “BCGs” to “BGCs” at all three indicated locations.

Lines 410-420: It is surprising that toxoflavin was not identified in this study, as the literature consistently reports toxoflavin as a common metabolite produced by B. glumae. It should also be noted that toxoflavin biosynthetic gene clusters are not typically identified directly by antiSMASH and may require targeted searches for known toxoflavin biosynthetic genes within the genome. The authors are encouraged to investigate the presence of these genes and, if possible, assess toxoflavin production under alternative culture conditions, such as different media or longer incubation periods on LB agar beyond 24 hours, which may enhance its expression.

Thank you for this insightful comment. We investigated the toxoflavin biosynthetic genes independently of antiSMASH using BLASTn, with the toxoflavin biosynthesis-associated locus spanning *toxJ* to *toxE* from *B. glumae* BGR-1 (NCBI accession NC_012721.2) as the reference query. A corresponding region was detected in all 16 isolates, including the four selected for metabolomic analysis, with >99% nucleotide identity and query coverage. We have added this approach to the Materials and Methods and revised the Discussion to report these findings without implying confirmed functional expression.

We did not assess toxoflavin production using alternative media or incubation periods beyond 24 h. However, we have expanded the Discussion with additional references on toxoflavin regulation and its production under different cultivation conditions. We acknowledge that these factors may contribute to differences between studies, but their effects were not directly assessed here and the reasons for non-detection remain unresolved.

The following text has been added to the Genome mining for specialized metabolite BGCs in *B. glumae* subsection of the Materials and Methods:

"The presence of toxoflavin biosynthetic genes was investigated independently of antiSMASH using BLASTn, with the toxoflavin biosynthesis-associated locus spanning *toxJ* to *toxE* from B. glumae BGR-1 (NCBI accession: NC_012721.2) [30] as the reference query."

The following text in the Discussion has been revised to clarify the targeted genomic findings and the possible explanations for metabolite non-detection:

The original text:

"A previous investigation of antimicrobial-active *B. glumae* isolates reported the detection of reumycin, bactobolin A, toxoflavin, and two potentially novel antibacterial compounds [20]. However, none of these metabolites were detected in the present study. This observation may reflect differences in cultivation conditions and/or strain-specific metabolic responses. The previous study cultivated isolates under nutrient-limited conditions and prolonged incubation [20], whereas the present study used nutrient-rich media and a shorter incubation period. Notably, all isolates examined in this study contained intact toxoflavin BGCs, indicating that the absence of toxoflavin and its derivative reumycin in the metabolomic analysis is unlikely due to a loss of biosynthetic capability. Instead, the absence of toxoflavin, reumycin, and bactobolin A, together with the enrichment of alternative metabolite classes observed here, likely reflects a condition-dependent metabolic variation."

Has been revised to:

"A previous investigation of antimicrobial-active *B. glumae* isolates reported the detection of toxoflavin, reumycin, bactobolin A and two potentially novel antibacterial compounds [21]. However, none of these metabolites were detected in the present study. Targeted BLASTn analysis identified a region corresponding to the toxoflavin biosynthesis-associated locus in all four selected isolates, with >99% nucleotide identity and query coverage. Nevertheless, sequence conservation alone does not establish functional expression or metabolite production. Toxoflavin biosynthesis and transport in *B. glumae* are regulated by ToxJ, whose expression is activated by quorum sensing, and the LysR-type regulator ToxR, which requires toxoflavin as a coinducer [43]. Differences in cultivation conditions may have influenced the metabolite profiles observed across studies. The previous study cultivated isolates on a nutrient-limited medium at 28 °C for four days [21], whereas the present study used LB agar at 37 °C for 24 h. Toxoflavin production has also been observed in *B. glumae* BGR1 grown on LB agar at 30 °C for 48 h [44], suggesting that nutrient-rich conditions do not inherently suppress its synthesis. These observations indicate that variables such as incubation temperature and duration may account for inter-study variations, though their direct influence was not investigated in the current study. Reumycin serves as a biosynthetic precursor of toxoflavin [31], suggesting that variation in pathway activity may influence the abundance and detectability of both compounds. Although bactobolin A was detected in the previous study [21], its biosynthetic basis and the factors regulating its production in *B. glumae* remain unclear."

Figures and Tables: Please improve the quality and resolution of the figures to enhance readability. In addition, the supplementary tables are very large and may be difficult for readers to navigate. Please consider revising their formatting, reducing unnecessary content where appropriate, or restructuring them to improve clarity and accessibility.

Thank you for this comment. We have checked the original figure files and confirm that their resolution meets the journal's requirements. The reduced quality in the review version may result from compression during PDF generation by the submission system. The original high-resolution files have been provided for publication.

We appreciate the concern regarding the size and navigability of the supplementary tables. These tables provide detailed results and raw data in accordance with the journal's data-sharing policy to support transparency and reproducibility, and facilitate future research, including secondary analyses and meta-analyses. We have therefore retained their full content to preserve the completeness of the dataset.

Reviewer #2:

By combining genome mining, screening of antibacterial activity in vitro and untargeted LC–MS/MS metabolomic profiling, Patarapuwadol et al. analyzed the antibacterial potential of 16 Burkholderia glumae isolates collected from Thailand. Genome mining using antiSMASH and DeepBGC identified several biosynthetic gene clusters associated with antibacterial activity in all isolates. To test antibacterial activity, the authors performed soft-agar overlay assays on Luria–Bertani medium. All 16 isolates displayed antibacterial activity against Acinetobacter baumannii, Escherichia coli, and Staphylococcus aureus. Four isolates exhibiting the strongest antibacterial activity were selected for further metabolomic analysis. The metabolic profiles of extracts from inoculated LB agar plates were compared to metabolic profiles of extracts from uninoculated LB agar plates. Sixty-eight putatively annotated antimicrobial metabolites of diverse chemical classes showed significant enrichment and were shared among the four isolates. The work highlight the potential of these B. glumae isolates as a source of antimicrobial compounds against important pathogens.

Comments:
1) Abstract. Line 38: Untargeted LC–MS/MS metabolomic profiling identified 68 putatively annotated antimicrobial-related metabolites showing significant enrichment (fold change > 2, p < 0.05) and shared among all four isolates.
In the abstract it is not stated that the authors compare the LC–MS/MS metabolomic profiling of extracts from inoculated agar plates to extracts from uninoculated agar plates (used as control). As a consequence, this sentence is difficult to interpret. The employed methodology should be briefly described in the abstract for ease of understanding.

Thank you for this comment. We have revised the Abstract to explicitly state that LC–MS/MS profiling compared extracts from inoculated LB agar plates with extracts from uninoculated LB agar plates used as medium controls. This clarification makes the basis of the reported metabolite enrichment clear.

The original text:

"Untargeted LC–MS/MS metabolomic profiling identified 68 putatively annotated antimicrobial-related metabolites showing significant enrichment (fold change > 2, p < 0.05) and shared among all four isolates."

Has been revised to:

"Untargeted LC–MS/MS profiling of extracts from inoculated and uninoculated LB agar plates identified 68 putatively annotated antimicrobial-related metabolites shared among all four isolates and significantly enriched relative to the uninoculated controls (fold change > 2, *p* < 0.05)."

We have also added the following clarification to the LC–MS/MS-based metabolite profiling subsection of the Materials and Methods.

"Uninoculated LB agar plates prepared without bacterial inoculation and incubated under the same conditions were used as medium controls and subjected to the same collection, extraction, and LC–MS/MS procedures as the inoculated samples."

2) Abstract. Line 42: Macrolactams represented the most frequently detected metabolite class, whereas triterpene saponins exhibited the highest enrichment relative to uninoculated controls. Same comment as above.

Thank you for this comment. As also requested by Reviewer 1, we have revised "uninoculated controls" to "control LB agar without bacterial inoculation." We have additionally clarified in the preceding sentence that LC–MS/MS profiling compared extracts from inoculated and uninoculated LB agar plates.

The original text:

"Macrolactams represented the most frequently detected metabolite class, whereas triterpene saponins exhibited the highest enrichment relative to uninoculated controls."

Has been revised to:

"Macrolactams represented the most frequently detected metabolite class, whereas triterpene saponins exhibited the highest enrichment relative to control LB agar without bacterial inoculation."

3) Abstract. Line 44: Interestingly, known B. glumae metabolites, such as ovoflavin, reumycin, and bactobolin A were not detected, suggesting that nutrient-rich cultivation conditions shifted expression toward alternative, putatively novel biosynthetic pathways.
To my understanding toxoflavin, reumycin and bactobolin A have been detected during cultivation in rich-media. Besides nutrient limitation, temperature and culture density also play an important role in the production of these metabolites. Therefore, the absence of these compounds could be attributed to different factors (as it is mentioned in the discussion section). I suggest to change this sentence.
Moreover, a brief description of the cultivation conditions and medium used in this work could be added in the Abstract for clarity.

Thank you for this comment. We agree that the non-detection of these metabolites cannot be attributed specifically to nutrient-rich cultivation conditions. We have removed this inference and specified the medium, temperature, and incubation duration in the Abstract.

The original text:

"Interestingly, known *B. glumae* metabolites, such as toxoflavin, reumycin, and bactobolin A, were not detected, suggesting that nutrient-rich cultivation conditions shifted expression toward alternative, putatively novel biosynthetic pathways."

Has been revised to:

"Toxoflavin, reumycin, and bactobolin A were not detected in extracts from cultures grown on LB agar at 37 °C for 24 h."

4) LC–MS/MS-based metabolite profiling, Line 180.
A sentence mentioning the use of uninoculated LB agar plates to prepare organic extracts as controls for LC–MS/MS metabolomic profiling should be mentioned in this section.

Thank you for this comment. We have added a sentence to the LC–MS/MS-based metabolite profiling subsection clarifying that uninoculated LB agar plates were used as medium controls and subjected to the same incubation, collection, extraction, and LC–MS/MS procedures as the inoculated samples.

The following sentence has been added:

"Uninoculated LB agar plates prepared without bacterial inoculation and incubated under the same conditions were used as medium controls and subjected to the same collection, extraction, and LC–MS/MS procedures as the inoculated samples."

5) Table 1, Page 13: I suggest to use a bar plot for displaying these results (to facilitate visualization).

Thank you for this suggestion. We have replaced Table 1 with a grouped bar plot to facilitate comparison of antibacterial activity across isolates and indicator strains. The numerical results have been retained in a supplementary table for reference.

6) Metabolomic analysis of selected B. glumae isolates. Line 314.
Please add that the metabolic profiles obtained from extracts of inoculated LB agar plates were compared to metabolic profiles obtained from extracts of uninoculated LB agar plates.

Thank you for this comment. We have revised the opening of this Results subsection to clarify that LC–MS analysis was performed on extracts from inoculated LB agar cultures and that the resulting metabolomic profiles were compared with those of extracts from uninoculated LB agar plates used as medium controls.

The original text:

“Untargeted LC–MS analysis was performed on three biological replicates of the four selected *B. glumae* isolates exhibiting the strongest antibacterial activity.”

Has been revised to:

“Untargeted LC–MS analysis was performed on extracts from three biological replicates of LB agar cultures of each of the four selected *B. glumae* isolates exhibiting the strongest antibacterial activity. The resulting metabolomic profiles were compared with those of extracts from uninoculated LB agar plates used as medium controls.”

7) Discussion. Line 406: Although this study did not establish direct links between specific metabolites and biosynthetic gene clusters, the consistency between the genome mining data and metabolomic analyses supports the prediction that B. glumae has the capacity to produce structurally diverse antibacterial metabolites.
A Table combining (and summarizing) the genome mining data (prediction of operons, genes) and the metabolic analyses is missing and would be very useful to analyze the consistency of the results.

Thank you for this helpful suggestion. We have added Table 2, “Summary of genome-mining predictions and metabolomic findings in the four selected *Burkholderia glumae* isolates,” to facilitate comparison of the genomic and metabolomic results. We have also added a corresponding paragraph to the “Metabolomic analysis of selected *B. glumae* isolates” Results subsection. These additions summarize potential class-level correspondences, including the detection of the toxoflavin biosynthesis region despite the non-detection of toxoflavin, while clarifying that direct links between individual BGCs and metabolites remain unestablished.

The following paragraph has been added:

“A comparison of genome-mining predictions and metabolomic findings is presented in Table 2. antiSMASH identified terpene-, polyketide-, and NRPS-associated regions in all four selected isolates, alongside metabolomic features putatively assigned to terpene-related, macrolactam, and peptide-related classes. Targeted BLASTn analysis also detected a region corresponding to the toxoflavin biosynthesis-associated locus in all four isolates with >99% nucleotide identity and query coverage, although toxoflavin was not detected. The biosynthetic origins of the alkylquinoline-related and remaining metabolomic features were unresolved. These comparisons summarize potential class-level correspondences without establishing direct links between individual BGCs and metabolites.”

8) Discussion. Line 410: A previous investigation of antimicrobial-active B. glumae isolates reported the detection of reumycin, bactobolin A, toxoflavin, and two potentially novel antibacterial compounds [20].
Please expand the discussion (by citing more studies) about factors influencing the production of these metabolites in B. glumae.

Thank you for this suggestion. We have revised the Discussion to incorporate additional studies on the regulation of toxoflavin biosynthesis and the potential influence of cultivation conditions on its production. We have also explained the precursor relationship between

reumycin and toxoflavin and acknowledged the limited understanding of bactobolin A biosynthesis and regulation in *B. glumae*. These additions provide further context for interpreting metabolite non-detection while acknowledging that the underlying reasons remain unresolved.

The original text:

"A previous investigation of antimicrobial-active *B. glumae* isolates reported the detection of reumycin, bactobolin A, toxoflavin, and two potentially novel antibacterial compounds [20]. However, none of these metabolites were detected in the present study. This observation may reflect differences in cultivation conditions and/or strain-specific metabolic responses. The previous study cultivated isolates under nutrient-limited conditions and prolonged incubation [20], whereas the present study used nutrient-rich media and a shorter incubation period. Notably, all isolates examined in this study contained intact toxoflavin BGCs, indicating that the absence of toxoflavin and its derivative reumycin in the metabolomic analysis is unlikely due to a loss of biosynthetic capability. Instead, the absence of toxoflavin, reumycin, and bactobolin A, together with the enrichment of alternative metabolite classes observed here, likely reflects a condition-dependent metabolic variation."

Has been revised to:

"A previous investigation of antimicrobial-active *B. glumae* isolates reported the detection of toxoflavin, reumycin, bactobolin A and two potentially novel antibacterial compounds [21]. However, none of these metabolites were detected in the present study. Targeted BLASTn analysis identified a region corresponding to the toxoflavin biosynthesis-associated locus in all four selected isolates. Nevertheless, sequence conservation alone does not establish functional expression or metabolite production. Toxoflavin biosynthesis and transport in *B. glumae* are regulated by ToxJ, whose expression is activated by quorum sensing, and the LysR-type regulator ToxR, which requires toxoflavin as a coinducer [43]. Differences in cultivation conditions may have influenced the metabolite profiles observed across studies. The previous study cultivated isolates on a nutrient-limited medium at 28 °C for four days [21], whereas the present study used LB agar at 37 °C for 24 h. Toxoflavin production has also been observed in *B. glumae* BGR1 grown on LB agar at 30 °C for 48 h [44], suggesting that nutrient-rich conditions do not inherently suppress its synthesis. These observations indicate that variables such as incubation temperature and duration may account for inter-study variations, though their direct influence was not investigated in the current study. Reumycin serves as a biosynthetic precursor of toxoflavin [31], suggesting that variation in pathway activity may influence the abundance and detectability of both compounds. Although bactobolin A was detected in the previous study [21], its biosynthetic basis and the factors regulating its production in *B. glumae* remain unclear."
